# Gut Bacteria Missing in Severe Acute Malnutrition, Can We Identify Potential Probiotics by Culturomics?

**DOI:** 10.3389/fmicb.2017.00899

**Published:** 2017-05-23

**Authors:** Maryam Tidjani Alou, Matthieu Million, Sory I. Traore, Donia Mouelhi, Saber Khelaifia, Dipankar Bachar, Aurelia Caputo, Jeremy Delerce, Souleymane Brah, Daouda Alhousseini, Cheikh Sokhna, Catherine Robert, Bouli A. Diallo, Aldiouma Diallo, Philippe Parola, Michael Golden, Jean-Christophe Lagier, Didier Raoult

**Affiliations:** ^1^URMITE, Aix Marseille Université, UM63, Centre National de la Recherche Scientifique 7278, IRD 198, Institut National de la Santé Et de la Recherche Médicale 1095, IHU—Méditerranée InfectionMarseille, France; ^2^Laboratoire de Microbiologie, Département de Biologie, Université Abdou Moumouni de NiameyNiamey, Niger; ^3^Département d'Epidémiologie des Affections Parasitaires, Faculté de Médecine, Université des Sciences, des Techniques et Technologies de BamakoBamako, Mali; ^4^Service de Médecine Interne et Générale, Hôpital de NiameyNiamey, Niger; ^5^Unité de Recherche sur les Maladies Infectieuses et Tropicales Emergentes IRD 198, Centre National de la Recherche Scientifique 7278, Aix-Marseille UniversitéDakar, Senegal; ^6^Department of Medicine and Therapeutics, University of AberdeenAberdeen, United Kingdom

**Keywords:** severe acute malnutrition, kwashiorkor, gut microbiota, culturomics, metagenomics, probiotics, *Methanobrevibacter smithii*, *Streptococcus gallolyticus*

## Abstract

Severe acute malnutrition is the world-leading cause of children under-five's death. Recent metagenomics studies have established a link between gut microbiota and severe acute malnutrition, describing an immaturity with a striking depletion in oxygen-sensitive prokaryotes. Amoxicillin and therapeutic diet cure most of the children with severe acute malnutrition but an irreversible disruption of the gut microbiota is suspected in the refractory and most severe cases. In these cases, therapeutic diet may be unable to reverse the microbiota alteration leading to persistent impaired development or death. In addition, as enteric sepsis is a major cause of death in this context, identification of missing gut microbes to be tested as probiotics (live bacteria that confer a benefit to the host) to restore rapidly the healthy gut microbiota and prevent the gut pathogenic invasion is of foremost importance. In this study, stool samples of malnourished patients with kwashiorkor and healthy children were collected from Niger and Senegal and analyzed by culturomics and metagenomics. We found a globally decreased diversity, a decrease in the hitherto unknown diversity (new species isolation), a depletion in oxygen-sensitive prokaryotes including *Methanobrevibacter smithii* and an enrichment in potentially pathogenic *Proteobacteria, Fusobacteria* and *Streptococcus gallolyticus*. A complex of 12 species identified only in healthy children using culturomics and metagenomics were identified as probiotics candidates, providing a possible, defined, reproducible, safe, and convenient alternative to fecal transplantation to restore a healthy gut microbiota in malnourished children. Microbiotherapy based on selected strains has the potential to improve the current treatment of severe acute malnutrition and prevent relapse and death by reestablishing a healthy gut microbiota.

## Introduction

Undernutrition is the worldwide leading cause of mortality for children under 5 years of age accounting for 1–6 million deaths every year (WHO | Levels trends in child mortality, 2015). Moreover, severe acute malnutrition (SAM) affects 20 million children, mostly from developing countries of sub-Saharan Africa, Central America and South Asia (UNICEF Nutrition Section et al., 2007). The World Health Organization (WHO) defines SAM using the anthropometric indicators of mid-upper arm circumference (MUAC) <115 mm, weight-for-height *z*-score (WHZ) < −3*z*-score and/or bilateral oedema (WHO and UNICEF, 2009). Chronic malnutrition, which has an even higher prevalence, is defined in terms of height for age and is the subject of intense investigation of “environmental enteropathy” whereby the upper intestine shows pathological lesions in children living in the tropics; these are thought to be related to chronic bacterial contamination from the environment and be causally related to poor growth in height. Non-oedematous SAM, erstwhile termed marasmus, has an incidence several times superior to that of the prevalence, with the average duration of illness of about 4 months. Oedematous malnutrition, which is now the definition of kwashiorkor and marasmic-kwashiorkor, has a much lower prevalence in surveys because it is an acute illness with a short history so that the number of cases presenting to medical facilities far exceeds those found in the community; for this reason, the importance of oedematous malnutrition has been underestimated.

Oedematous SAM is referred to in the Bible and was described in Europe in the nineteenth century as idiopathic oedema, in Mexico and Viet-Nam, and later was named kwashiorkor by Williams in 1933 (Williams, 1933). There are no convincing animal models of kwashiorkor with the exception of primates given the children's diet in Uganda (Coward and Whitehead, 1972). The etiology of oedematous malnutrition as well as other types of oedema is still disputed (Golden, 2015). The presence of oedema in SAM has been associated with the absence of breastfeeding, lower household dietary diversity score and lower fish, nuts, dairy products, green leafy vegetables, and fresh fruits consumption (Rytter et al., 2015). Recently, gut microbiota was considered an instrumental factor in kwashiorkor pathogenesis (Smith et al., 2013). Exploration of the gut microbiota of malnourished children using metagenomics and molecular approaches demonstrated that the colonic microbiota has lost diversity (Smith et al., 2013). Strictly anaerobic species are reduced; in particular, *Methanobrevibacter smithii*, the most oxygen-sensitive prokaryote of the human gut, was totally absent. The loss of anaerobic diversity was associated with a high redox potential and a relative enrichment in aerobic species (Million et al., 2016). On the other hand, diarrhea, high intestinal inflammation, low concentration of fecal butyrate and propionate, and high systemic inflammation have been related to mortality in SAM. However, these relations were not mediated by the presence of intestinal pathogens suggesting the loss of a previously unknown gut protective factor (Attia et al., 2016). We previously proposed that the healthy mature predominantly anaerobic gut microbiota (HMAGM) is the protective factor capable of stopping diarrhea, decreasing intestinal inflammation, producing butyrate and propionate (shown to decrease systemic inflammation) and controlling intestinal pathogens (Million et al., 2017b).

The treatment of uncomplicated SAM has been revolutionized by the use of Ready-to-Use-Therapeutic-Food (RUTF) in an outpatient setting (UNICEF Nutrition Section et al., 2007); RUTF has the same nutritional profile as F100 (with iron) that is the standard treatment of all children with SAM, but is presented as a paste with a very low water activity, so that it is safe to give at home. Antibiotics have been routinely given to malnourished children since the 1960s and their administration is recommended in all standard treatment protocols. With modern treatments, the mortality rate has fallen from about 40% to about 5% when using updated protocols (Trehan et al., 2013; Million et al., 2017a). However, the relapse rate remains disappointingly high and long-term follow-up of ex-patients shows a continued high mortality rate after seemingly successful treatment.

In order to improve the current treatment protocol and prevent relapse and deaths of kwashiorkor patients, a restoration of the gut microbiota of malnourished patients could improve their health outcome. Fecal transplant is a promising technique to restore a healthy microbiota, and it has been very successful in controlling or eliminating colonic pathogens under specific circumstances (van Nood et al., 2013); however its routine use is not established (Khoruts et al., 2015). This is partly due to questions of quality control in terms of unpredictable variation in the donors' microbiota. The ideal would be to be able to define and reliably reproduce a known and effective mixed fecal biota by culturing those symbionts that are missing from diseased patients but present in healthy subjects. The main objective of this study is to identify those organisms that are likely candidates for children with kwashiorkor.

A study examining the microbiota of twins' discordance for kwashiorkor showed differences between the resident organisms of diseased and healthy twins (Smith et al., 2013). The organisms present in the ill twins were cultured and introduced to gnotobiotic mice; the mice became ill and had significant weight loss. They did not develop features typical of the human condition, however, as efforts to recreate an animal model of kwashiorkor in rodents have been universally unsuccessful. Nevertheless, this study indicates that changing the resident microbiota of malnourished patients either by fecal transplant or preferably by specific species used as probiotics could be beneficial. The “missing” microbes in the lower intestine of patients with kwashiorkor have been initially identified (Million et al., 2017b) and comprise mainly anaerobes. As utilization of the missing repertoire of probiotics requires viable isolated strains, these can only be obtained by culturomics. The ability for high throughput culture of such gut microbiota has been demonstrated (Lagier et al., 2016). The difference between metagenomics, which demonstrates which organisms were present in the colon, and culturomics which specifically identifies viable live organisms is crucial. It is likely that many of the DNA sequences identified by metagenomics come from organisms that are already dead and thus would not be candidates for isolation and multiplication, this clearly is not the case with culturomics.

The Food and Agriculture Organization of the United Nations and the World Health Organization define probiotics as live micro-organisms which, when administered in adequate amounts, confer a health benefit on the host (Schlundt, 2001). We consider that the following characteristics are desirable in a probiotic imitating the neonatal gut microbiota transplantation from the mother to the infant: persistently viable in a healthy gut environment, do not produce any toxic product (particularly lithocholate), symbiotic with healthy resident microbiota, producing butyrate and/or propionate and anti-pathogenic bacteriocins, and promotes gut environment characteristic of good health (Million et al., 2017b). For instance, the colon is normally strictly anaerobic whereas in severe acute malnutrition, the colonic lumen becomes oxidizing (Million et al., 2016). Resistance to physiological oxidative and nitrosative stress of the stomach and bile salts were not required as most of the healthy mature anaerobic gut microbiota (HMAGM) members didn't fulfill these criteria and have been used successfully as probiotics in *C. difficile* infection (Petrof et al., 2013). The objective of this study was to identify potential bacteria which met these criteria, were present in healthy children and deficient in children with severe acute malnutrition.

In this study, we described the gut microbiota of patients with kwashiorkor and healthy children using both metagenomics and culturomics to identify prokaryote candidate probiotics that could restore a healthy gut microbiota in malnourished children. The complementarity between metagenomics and culturomics (Lagier et al., 2012) makes these two techniques particularly adapted to this study, enabling a better exploration of the fecal samples and the possibility to make probiotics available for further study.

## Materials and methods

### Population and samples

**Figure 1:**
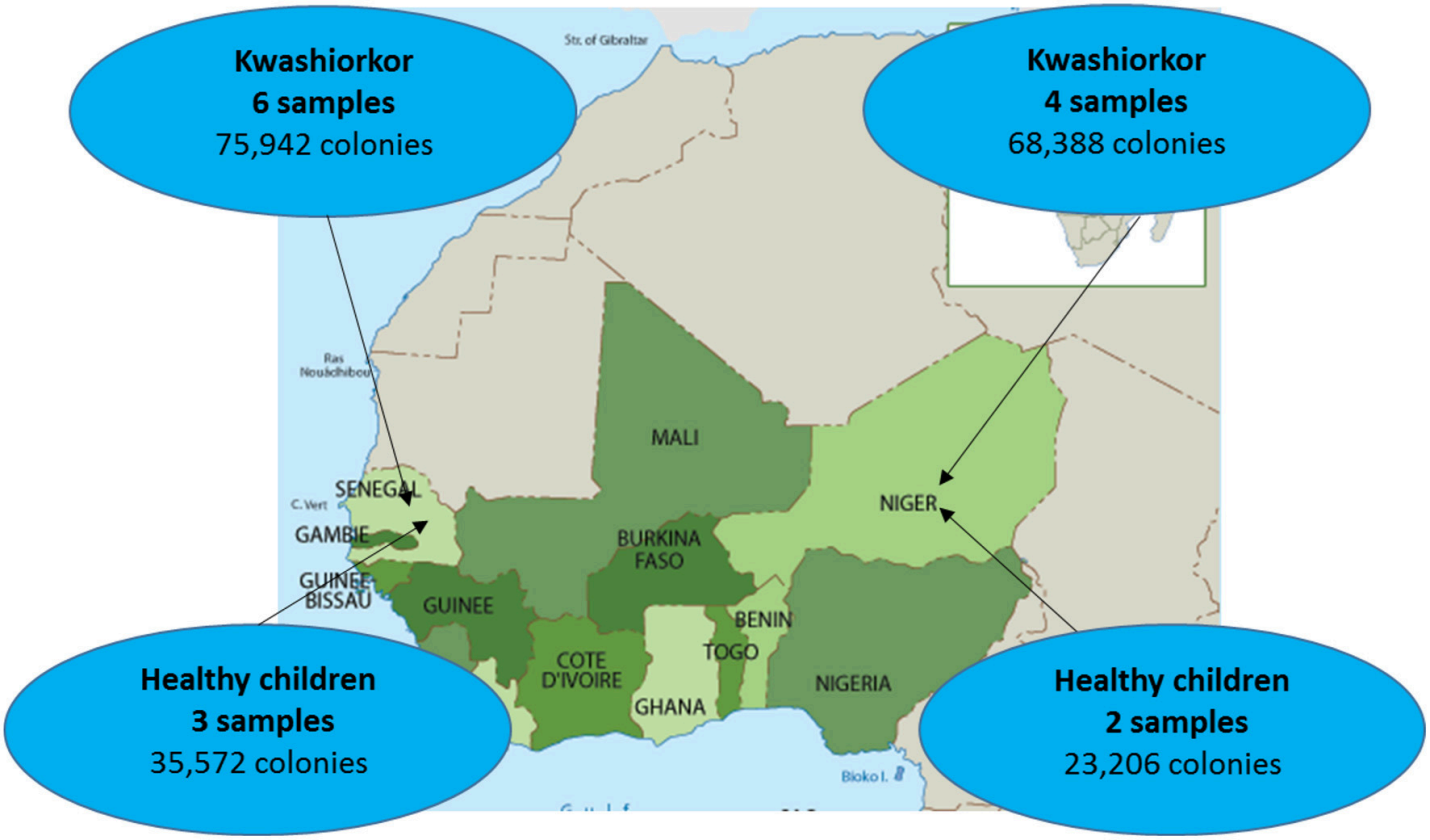
**Sample repartition according to geographic origin**. Four samples were collected from patients with kwashiorkor in Niger while six samples were collected from patients with kwashiorkor in Senegal. As for controls, stool samples were collected from three healthy children from Senegal and two healthy children from Niger.

Ten severely undernourished children with nutritional oedema (kwashiorkor) and five healthy children without any criteria of malnutrition (no wasting, no stunting, and not underweight), according to the 2009 WHO criteria (WHO and UNICEF, 2009), were recruited in Senegal and Niger (Supplementary Table 1). Two African centers were included to test geographical generalization (Figure 1). The aforementioned number of samples was chosen because of the time requirement for culturomics analysis of each sample (12,000 colonies isolated by 18 culture conditions after inoculation at day 1, 3, 7, 10, 15, 21, and 30 of the pre-incubated fecal samples corresponding to a 6 weeks' protocol for each sample). More cases than controls were included since we favored the characterization of the gut microbiota of malnourished children. Six and four cases were included in the Campus International UCAD/IRD of Hann, Dakar, Senegal and in the Pediatrics emergency room of the National Hospital of Niamey, Niger, respectively. Three and two controls of same sex and similar age were recruited by a snowball approach in Dakar and Niamey, respectively. All the parents of the children involved in this study gave an informed consent for the participation of their children. Since most of the parents were illiterate, a verbal consent procedure was adopted for a homogeneity purpose. Local authorities approved the study and were also present during inclusions. This procedure was approved by the Ethic Committee of the Institute Fédératif de Recherche 48 since it was authorized by the French Bioethics Law N° 2004-800 (06/08/2004) for non-epidemiological studies.

### Microbial culturomics: high-throughput bacterial culture

#### High-throughput bacterial culture

**Figure 2:**
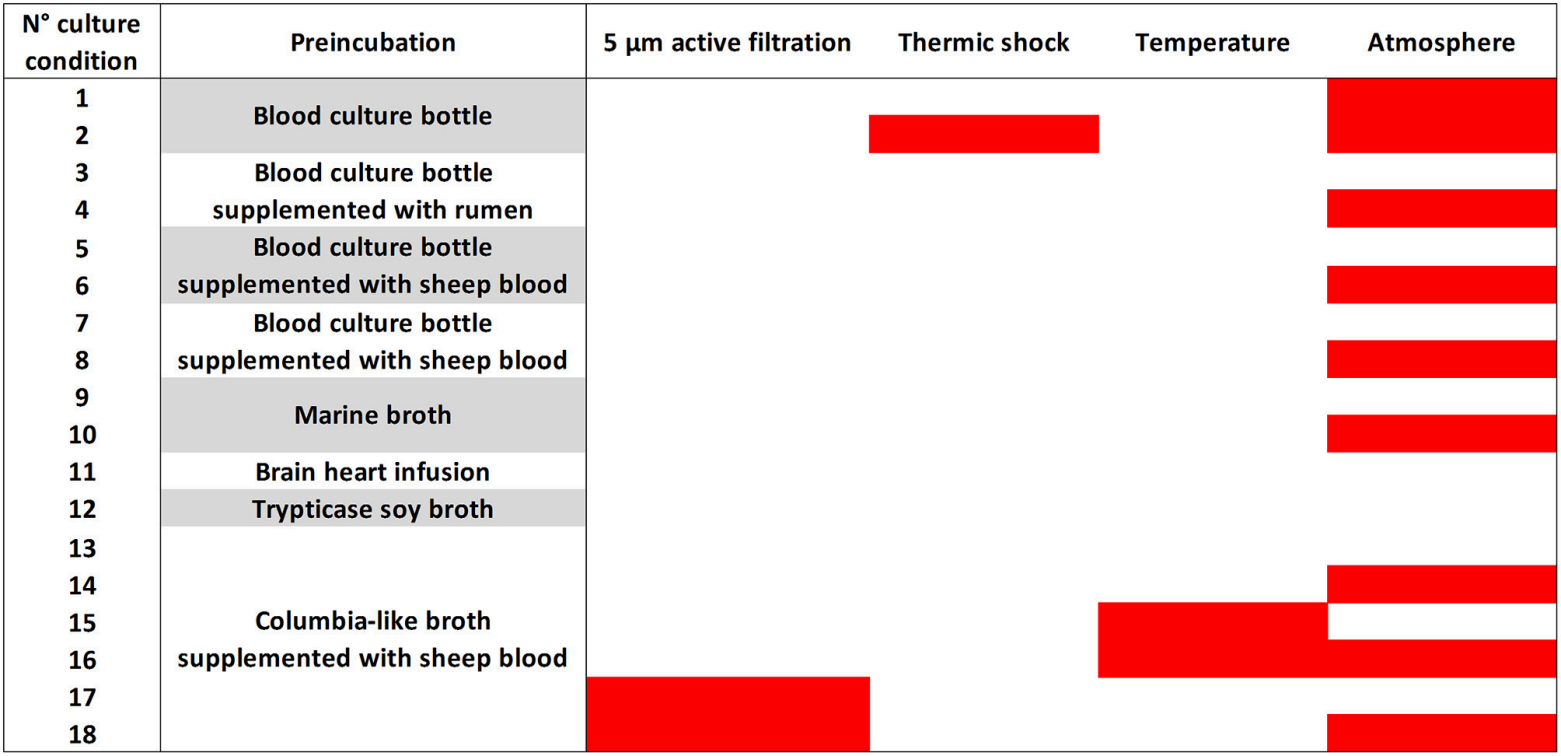
**The 18 culture conditions of standardized culturomics**. The 18 culture conditions are here represented according to the preincubation liquid medium, mode of treatment of the stool sample, temperature, and atmosphere. The red bars represent in the third column an active filtration (5 μm) applied to the stool sample, in the fourth column a thermal shock (80°C during 20 min) applied to the stool sample, in the fifth column a 28°C incubation temperature and in the sixth column an anaerobic atmosphere of incubation. No coloration represents in the third column no active filtration applied to the stool sample, in the fourth column no thermal shock applied to the stool sample, in the fifth column a 37°C incubation temperature and in the sixth column an aerobic atmosphere of incubation.

Samples were analyzed using 18 culture conditions (Figure 2) with identification of 12,000 colonies by sample as described previously (Lagier et al., 2015b). For each condition, 1 g of each sample was diluted in 9 mL of PBS (ThermoFisher Scientific, Illkirch, France); the suspension was then inoculated in liquid medium and incubated at the chosen temperature atmosphere. At day 1, 3, 7, 10, 15, 21, and 30, 10-fold serial dilutions of the culture were inoculated on 5% sheep blood Agar (Becton Dickinson, Le Pont de Claix, France). A subculture of the resulting colonies was performed for purification. The colonies were then identified using Matrix Assisted Laser Desorption/ Ionization Mass Spectrometry (MALDI-TOF/MS; Seng et al., 2009, 2013).

#### High-throughput bacterial identification: MALDI-TOF/MS

The MALDI-TOF MS identification was performed with a microflex from Bruker Daltonics (Bremen, Germany) according to the manufacturer's instructions. Each colony was deposited in duplicate on a 96 MSP microplate and covered with 2 μL matrix solution. The solution was made of saturated α-cyano-4-hydroxycinnamic acid, 50% acetonitrile and 2.5% trifluoroacetic acid. Each obtained spectrum was matched against the 7562 spectra of Bruker's and the Laboratory of La Timone's home database (as of January 2016). A score >1.9 allowed identification at the species level (Seng et al., 2009).

#### Identification of strains unidentified by MALDI-TOF/MS

DNA from the unidentified colonies (MALDI-TOF score <1.9) was extracted using EZ1 DNA Tissue Kit from Qiagen in an automated EZ1 advanced instrument according to the manufacturer's instructions. The DNA was amplified with primers FD1 and RP2 targeting all bacteria at an annealing temperature of 52°C. The amplified product was purified on a 96-well-purification plate and then re-amplified using the BigDye Terminator v1.1 cycle sequencing kit (Qiagen) at an annealing temperature of 52°C with primers FD1, RP2, 536F, 536R, 800F, 800R, 1050 F, and 1050R (Supplementary Table 2). The product was purified and analyzed using an ABI PRISM 3130x Genetic Analyzer (Applied Biosystems). The resulting sequences were analyzed using the software ChromasPro and compared to the NCBI database with the BLAST software. Sequences with a similarity percentage under 98.65 or 95% (Kim et al., 2014) were identified as new species or new genera, respectively, and described according to the taxonogenomics concept (Fournier et al., 2015).

### High throughput sequencing

Total DNA was extracted from the stool samples using two protocols (Dridi et al., 2009). The first one is based on a physical lysis with glass powder followed by an overnight chemical lysis proteinase K. The resulting solution is washed and eluted according to the Macherey-Nagel DNA Tissue extraction kit (Dridi et al., 2009). For the second protocol, glycoprotein lysis and deglycosylation steps were added to the first protocol (Angelakis et al., 2016). Sequencing of the resulting DNA from these two protocols was performed targeting the V3–V4 regions of the 16S rRNA gene as previously described (Million et al., 2016). All reads from the two protocols were grouped and clustered with a threshold of 97% identity to obtain Operational Taxonomic Units (OTU). All OTUs made of <20 reads were removed. Remaining OTU were blasted against SILVA123 and assigned to a species if they matched one with at least 97% identity. OTU not assigned to any species were considered “unidentified.” As several OTUs matched identical species, the total number of identified species + the number of unidentified OTU was expected to be smaller than the total number of OTUs.

### Definition of the hitherto unknown diversity

As mentioned above, microbial culturomics give an unprecedented opportunity to access and quantify the hitherto unknown microbial diversity. Indeed, 16S targeted metagenomics gives us the possibility to assess and quantify richness and abundance of previously undescribed bacteria, evidencing that most of the richness, and diversity of the gut microbiota was unknown to date (Hayashi et al., 2002). We defined the “hitherto unknown diversity” as the number of new species added to the number of species not previously known from the human gut by sample for culturomics analysis and as the number of unidentified OTU for metagenomics analysis.

### Diversity assessment

β-diversity (Anderson et al., 2011) is a measure of biodiversity comparing the species diversity between ecosystems along environmental gradients. This involves comparing the number of taxa that are unique to each ecosystem. To assess the microbial diversity by culturomics, we defined the “unique microbiota” as the group of species found in only one child of each group. Accordingly, we calculated the unique/total microbiota richness (the U/T ratio) of each children group (kwashiorkor or controls) to assess this β-diversity. This ratio was not expected to be biased by the different sample sizes between the two groups. Accordingly, higher unique/total microbiota richness (increased U/T ratio) represents a higher diversity. For the metagenomics results, the diversity was estimated by calculating Shannon indexes for all the species, aerotolerant and anaerobic species using the following formula: H′ = ∑${\text{p}}_{\text{i}}^{*}$log_2_p_i_ where pi is the proportion of each species in the sample which diversity is being estimated (Spellerberg and Fedor, 2003. Metagenomics and culturomics results were compared in order to highlight the complementarity between these two methods. The hitherto unknown global and strict anaerobic diversity of cases and controls was compared using a culturomics approach. The ultimate goal of this study was to determine which microbes were missing in kwashiorkor patients. The species identified by both culturomics and metagenomics for each group of samples were compared and the species specific to healthy children were identified and their biological interest investigated to assess their potential usefulness as probiotics in SAM.

### Statistical analysis

For each quantitative analysis, normality was tested using either D'Agostino-Pearson or Kolmogorov-Smirnov tests for a small number of samples. Two-tailed unpaired student's *t*-test or Mann-Whitney test were used according to normality (Gaussian distribution). Two-tailed exact Fisher test or uncorrected Chi-squared test were used to compare proportions. Barnard two-tailed test was used when the sample size was very small (Barnard, 1945). All statistical analyses were performed with SPSS v21.0 (IBM, Paris, France). Correction for false discovery rate was not necessary since this is an exploratory study (Rothman, 1990).

## Results

### Population and descriptive culturomics and metagenomics results

Ten patients with kwashiorkor and five controls were selected. Six patients and three controls were recruited in Senegal and four patients and two controls were recruited in Niger, so that the proportion of patients with kwashiorkor originated from Niger and Senegal was identical (67%). No significant difference was observed for the age and sex of the patients with kwashiorkor and controls (Table 1). All 15 samples were analyzed using 18 culture conditions. For patients with kwashiorkor, 144,330 colonies were isolated and tested using MALDI TOF MS with a mean of 14,433 colonies per sample [standard deviation (*SD*), 2,975]. For control samples, 59,578 colonies were tested using MALDI-TOF MS with an average of 11,916 ± 404 colonies per sample. A total of 335 species were isolated in kwashiorkor samples whereas 281 species were isolated in control samples. All 15 samples were analyzed by 16S rRNA gene-targeted metagenomics. A total of 2,933,416 and 1,842,831 reads were generated from the 10 kwashiorkor and 5 control samples, respectively.

**Table 1:** **Baseline characteristics**.

	**Kwashiorkor (*n* = 10)**	**Controls (*n* = 5)**	***P*-value**
Age (months, mean ±*SD*)	13.4 ± 17.8^a^	25.1 ± 7.6	0.20^b^
Sex (Female)	3/6 (50%)^a^	3/5 (60%)	0.99^c^
Oedema	10 (100%)	0 (0%)	
Weight (kg)	5.2 ± 0.8	12.2 ± 1.9	0.004^d^
Height (cm)	61.2 ± 3.8	89.0 ± 8.7	0.01^d^
WHZ	NR	−0.4 ± 0.25	
WAZ	NR	−0.13 ± 1.02	
HAZ	−4.0 ± 1.3^a^	0.5 ± 2.0	0.07^d^

SD, Standard deviation; WHZ, Weight-for height z-score; WAZ, Weight-for-age z-score; HAZ, Height-for-age z-score; NR, Not relevant in the presence of edema;

a Age and sex missing for four samples from Niger;

b Two-tailed Mann-Whitney test;

c Two-tailed Barnard test;

d *Two-tailed unpaired t-test*.

### Decreased diversity in patients with kwashiorkor

**Figure 3:**
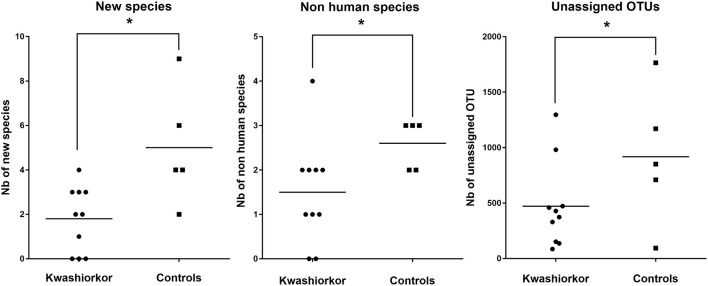
**Comparison of the Hitherto Unknown Diversity between patients with kwashiorkor and controls**. With culturomics, the hitherto unknown diversity is represented by the number of new species and the number of species not previously known in the human gut while with metagenomics, the hitherto unknown diversity is represented by the number of unassigned OTU. The hitherto unknown diversity was compared in kwashiorkor and control groups. A significant loss of the hitherto unknown diversity is observed in patients with kwashiorkor. *p < 0.05.

The U/T ratio (the ratio of species found in only one individual on the total number of species in the group, see Section Diversity Assessment) was significantly lower in the kwashiorkor group indicating a lower β-diversity [151/335 (45%) in kwashiorkor samples vs. 185/281 (66%) in the control group, uncorrected two-tailed chi squared test, *p* < 3 × 10^−7^, Table 2]. Forty-five species not known from the human gut were isolated from the kwashiorkor group (*n* = 10) including nine new species and nine new genera (Table 3), 15 are known but had not been previously found in humans and 12 are already known in humans but had not been previously found in the gut. In our group of five controls, we isolated 46 species unknown from the human gut including 26 new species (Table 3), among which eight new genera and one new family (*Neofamiliaceae* fam. nov.), 14 are known but had not been previously found in humans and six are already known in humans but had not been previously found in the gut. The hitherto unknown diversity assessed by culturomics was dramatically decreased in the kwashiorkor group (Table 4). However, the difference was significant only for new species (mean number of new species found by sample ± standard deviation, 1.8 ± 1.5 in kwashiorkor vs. 5.0 ± 2.6 in controls, unpaired two-tailed student's *t*-test, *p* = 0.009, Figure 3) and for previously known species but which had not been previously found in humans (1.5 ± 1.2 for kwashiorkor vs. 2.8 ± 0.4 for controls, two-tailed Mann Whitney test, *p* = 0.02, Table 4) suggesting a decreased α-diversity.

**Table 2:** **Culturomics highlights an altered diversity in kwashiorkor**.

**All samples**	**Kwashiorkor (*n* = 10)**	**Controls (*n* = 5)**	**Δ Diversity**	***P*-value**
All species^a^	151/335 (45%)	185/281 (66%)	+21%	2.5*10^−7^^c^
Anaerobic species^a^	43/111 (39%)	76/112 (68%)	+29%	0.00001^c^
Aerotolerant species^a^	108/224 (48%)	109/169 (64%)	+16%	0.001^c^
*Actinobacteria*^b^	47/335 (14%)	42/281 (15%)	+1%	0.74^c^
*Bacteroidetes*^b^	21/335 (6%)	21/281 (7%)	+1%	0.55^c^
*Firmicutes*^b^	208/335 (62%)	192/281 (68%)	+6%	0.1^c^
*Fusobacteria*^b^	3/335 (0.8%)	0/281 (0%)	−0.8%	0.32^d^
*Proteobacteria*^b^	56/335 (17%)	25/281 (9%)	−8%	0.004^c^

a Beta-diversity was assessed using the U/T ratio (U/T: Unique/Total).

b The diversity at the phylum level was assessed by the proportion in each phylum among the total number of species isolated.

c Uncorrected two-tailed Chi square test.

d *exact two-tailed Fisher test*.

*Δ Diversity = Diversity in controls − Diversity in kwashiorkor*.

*Global diversity, assessed by the U/T ratio (see Section Materials and Methods), is significantly decreased alongside the anaerobic and aerotolerant diversity in patients with kwashiorkor. Proteobacteria species were significantly enriched in kwashiorkor. No Fusobacteria species were isolated in controls*.

**Table 3:** **Putative new species according to their phylum, their tolerance to oxygen and their origin (Tidjani Alou et al., 2015a,b, 2016a,b,c,d,e,f,g, 2017; Alou et al., 2016a,b, 2017; Beye et al., 2016; Cimmino et al., 2016; Guilhot et al., 2016; Hadjadj et al., 2016; Seck et al., 2016; Traore et al., 2016a,b,c; Vicino et al., 2016; Pham et al., 2017)**.

**Species**	**Family**	**Oxygen tolerant**	**Sample of origin**	**16S rRNA accession number^a^**	**CSUR number^b^**	**DSMZ number^c^**
*Africanella massiliensis*^d^	*Ruminococcaceae*	No	Control	LT223700	P2538	DSM 102984
*Bacillus massilionigeriensis*	*Bacillaceae*	Yes	Control	LT161887	P2348	DSM 102112
*Bacillus mediterraneensis*	*Bacillaceae*	Yes	Control	LT161888	P2366	DSM 102091
*Bacillus phoceensis*	*Bacillaceae*	Yes	Control	LN881595	P2184	Pending
*Bacillus testis*	*Bacillaceae*	Yes	Control	LN827531	P1492	DSM 101190
*Bacillus touaregensis*	*Bacillaceae*	Yes	Control	LT223701	P2489	DSM 103460
*Brachybacterium massiliense*	*Dermabacteraceae*	Yes	Control	LN906631	P2240	DSM 101766
*Brevibacterium phoceense*	*Brevibacteriaceae*	Yes	Control	LN998064	P2230	Pending
*Clostridium massiliodielmoense*	*Clostridiaceae*	No	Control	LN998063	P2255	Pending
*Clostridium nigeriense*	*Clostridiaceae*	No	Control	LT161894	P2414	DSM 102218
*Enterobacter timonensis*	*Enterobacteriaceae*	Yes	Control	LN906632	P2201	DSM 101775
*Khelaifiabacterium massiliensis*^d^	*Clostridiaceae*	No	Control	LN850733	P1935	DSM 100591
*Lachnoclostridium massiliosenegalense*	*Lachnospiraceae*	No	Control	LT161890	P299	DSM 102084
*Lachnoclostridium touaregense*	*Lachnospiraceae*	No	Control	LT161895	P2415	DSM 102219
*Lagierella massiliensis*^d^	*Peptoniphilaceae*	No	Control	LN870299	P2012	DSM 100854
*Lascolabacillus massiliensis*^d^	*Porphyromonadaceae*	No	Control	LN827535	P1560	DSM 100190
*Massiliobacillus massiliensis*^d^	*Sporomusaceae*	No	Control	LT161896	P2411	DSM 102838
*Murdochiella massiliensis*	*Peptoniphilaceae*	No	Control	LN866998	P1987	DSM 100630
*Ndiopella massiliensis*^d^	*Peptoniphilaceae*	No	Control	LN866993	P1917	DSM 100643
*Neofamilia massiliensis*^e^	*Neofamiliaceae*	No	Control	LN866999	P1998	DSM 100639
*Niameyia massiliensis*^d^	*Lachnospiraceae*	No	Control	LN850735	P1909	DSM 100592
*Paenibacillus phoceensis*	*Paenibacillaceae*	Yes	Control	LN998053	P2238	DSM 101777
*Paenibacillus senegalomassiliensis*	*Paenibacillaceae*	Yes	Control	LN890284	P2144	Pending
*Paenibacillus touaregensis*	*Paenibacillaceae*	Yes	Control	LT223571	P2472	DSM 102801
*Peptoniphilus phoceensis*	*Peptoniphilaceae*	No	Control	LN881605	P2183	Pending
*Senegalia massiliensis*^b^	*Clostridiaceae*	No	Control	LN881608	P2130	Pending
*Anaerococcus rubiinfantis*	*Peptoniphilaceae*	No	Kwashiorkor	LN881592	P2032	DSM 101186
*Anaeromassilibacillus senegalensis*^d^	*Ruminococcaceae*	No	Kwashiorkor	LN866991	P1511	DSM 102594
*Anaerotruncus rubiinfantis*	*Ruminococcaceae*	No	Kwashiorkor	LN881593	P2276	DSM 101192
*Bacillus andreraoultii*	*Bacillaceae*	Yes	Kwashiorkor	LK021120	P1162	DSM 29078
*Bacillus niameyensis*	*Bacillaceae*	Yes	Kwashiorkor	LK985389	P1266	DSM 29725
*Bacillus rubiinfantis*	*Bacillaceae*	Yes	Kwashiorkor	LK021113	P1141	DSM 28615
*Desnuesiella massiliensis^d^*	*Clostridiaceae*	Yes	Kwashiorkor	LN846906	P1919	DSM 101500
*Flaviflexus massiliensis*	*Actinomycetaceae*	Yes	Kwashiorkor	LK985390	P1300	DSM 29058
*Inediibacterium massiliense*^d^	*Clostridiaceae*	No	Kwashiorkor	LN850734	P1907	DSM 100590
*Massilibacterium senegalense*^d^	*Bacillaceae*	Yes	Kwashiorkor	LN828943	P1510	DSM 100455
*Mediannikovella massiliensis*^d^	*Clostridiaceae*	No	Kwashiorkor	LN849776	P1934	DSM 100589
*Mobilicoccus massiliensis*	*Dermatophilaceae*	Yes	Kwashiorkor	LK985391	P1306	DSM 29065
*Nigerium massiliensis*^d^	*Propionibacteriaceae*	No	Kwashiorkor	LK985392	P1302	DSM 29084
*Olsenella massiliensis*	*Atopobiaceae*	No	Kwashiorkor	LN827536	P1476	DSM 100642
*Paenibacillus rubiinfantis*	*Paenibacillaceae*	Yes	Kwashiorkor	LN881603	P2076	DSM 101191
*Rubeoparvulum massiliense*^d^	*Bacillaceae*	No	Kwashiorkor	LN828926	P1473	DSM 100479
*Rubiinfantum massiliense*^d^	*Bacillaceae*	Yes	Kwashiorkor	LK985393	P2452	DSM 29059
*Tessaracoccus massiliensis*	*Propionibacteriaceae*	Yes	Kwashiorkor	LK985394	P1301	DSM 29060

a *EMBL/EBI accession number; sequence available on Genbank*.

b *CSUR, Collection de Souches de l'Unité des Rickettsies*.

c *DSMZ, Deutsche Sammlung von Mikroorganismen und Zellkulturen*.

d *New genus*.

e *New family*.

**Table 4:** **Comparison of the cultured gut bacterial diversity between children with kwashiorkor and control children**.

**Per sample**	**Kwashiorkor (*n* = 10)**	**Controls (*n* = 5)**	***P*-value**
**GLOBAL DIVERSITY (MEAN** ±***SD*****)**			
Nb of phyla	4.2 ± 0.6	3.8 ± 0.4	0.2^a^
Nb of genera	36 ± 7	34 ± 12	0.67^b^
Total Nb of species	90 ± 22	92 ± 20	0.82^b^
Nb of HG species	86 ± 21	85 ± 18	0.92^b^
Nb of species H but not G	1.2 ± 0.8	1.4 ± 0.9	0.75^a^
Nb of NH species	1.5 ± 1.2	2.8 ± 0.4	0.02^a^
Nb of new species	1.8 ± 1.5	5 ± 2.6	0.009^b^
**DIVERSITY BY PHYLUM [Nb SPECIES IN EACH PHYLUM (MEAN** ±***SD*****)]**			
*Firmicutes*	60 ± 18	68 ± 13	0.39^b^
*Proteobacteria*	11 ± 5	8 ± 7	0.3^b^
*Actinobacteria*	12 ± 4	11 ± 4	0.7^b^
*Bacteroidetes*	6.6 ± 4.2	5.4 ± 7.7	0.32^a^
*Fusobacterium*	0.5 ± 0.8	0 ± 0	0.21^b^
**DIVERSITY BY GENUS [Nb SPECIES IN EACH GENUS (MEAN** ±***SD*****)]**			
*Clostridium*	14 ± 6	18 ± 9	0.27^b^
*Bacillus*	9 ± 5	10 ± 3	0.6^b^
*Paenibacillus*	7 ± 1	8 ± 2	0.23^b^
*Streptococcus*	6 ± 1	5 ± 2	0.39^b^
*Staphylococcus*	6 ± 3	4 ± 2	0.10^b^
*Lactobacillus*	2.1 ± 2.1	1.6 ± 2	0.67^b^
*Bifidobacterium*	2.4 ± 1.7	0.8 ± 1.3	0.09^b^

*SD, Standard deviation; HG species: species previously isolated in the human gut, species H but not G: species previously isolated in humans but not in the gut, NH species: species not previously isolated in humans*.

a *Two-tailed Mann-Whitney test*.

b *Two-tailed unpaired t-test*.

*The unknown diversity is lower in the gut microbiota of patients with kwashiorkor (significantly less new species and NH species isolated per sample of kwashiorkor)*.

With metagenomics, calculating the mean Shannon Index ± *SD* by group showed that the global α-diversity was decreased in kwashiorkor even if not significantly (3.2 ± 0.8 vs. 3.8 ± 0.8 in controls, two-tailed student's *t*-test, *p* = 0.19, Table 5), consistent with previous studies (Subramanian et al., 2014). The hitherto unknown diversity assessed was consistently and significantly decreased in kwashiorkor as unidentified OTUs were lower in the kwashiorkor group (Figure 3). Accordingly, at the prokaryotic level, only 5% of all reads in the kwashiorkor group were not assigned vs. 26% in control patients (percentage of reads unassigned at the prokaryotic level ± *SD*, 0.05 ± 0.02 for kwashiorkor vs. 0.26 ± 0.22 for controls, *p* = 0.009).

**Table 5:** **Metagenomics evidenced a decreased fecal anaerobic diversity in kwashiorkor**.

**Per sample**	**Kwashiorkor (*n* = 10)**	**Controls (*n* = 5)**	***P*-value^a^**
Global diversity^b^ (mean ±*SD*)	3.2 ± 0.8	3.8 ± 0.8	0.19
Aerotolerant diversity^b^	2.0 ± 0.8	1.3 ± 0.6	0.1
Anaerobic diversity^b^	1.0 ± 1.0	3.1 ± 1.5	0.02

*SD, Standard deviation*.

a *Two-tailed unpaired t-test*.

b *Shannon indexes were calculated (see Section Materials and Methods) for each sample. Using metagenomics, only anaerobic diversity was significantly decreased in kwashiorkor*.

For the purposes of identification of potential probiotic species, we considered all of the bacterial species that were identified both by culturomics and metagenomics in the controls samples but not in the kwashiorkor samples.

### Loss of anaerobic species in patients with kwashiorkor

#### Anaerobic species are lost in patients with kwashiorkor

According to the “culturomics” results, both aerotolerant and anaerobic β-diversities were significantly lower in kwashiorkor (Table 4) but the decrease of anaerobic β-diversity (−29%, U/T ratio, 43/111 (39%) in kwashiorkor vs. 76/112 (68%) in controls, *p* = 0.00001) was larger than the decrease in aerotolerant β-diversity [−16%, 108/224 (48%) in kwashiorkor vs. 109/169 (64%) in controls, *p* = 0.001]. The difference was significant (Δ aerotolerant β-diversity/Δ anaerobic β-diversity, One-sample test for binomial proportion, normal-theory method, *p* < 10^−7^).

The metagenomics analysis showed a non-significant increased aerotolerant α-diversity (Shannon index ± standard deviation, 2.07 ± 0.8 in kwashiorkor vs. 1.35 ± 0.6 in controls, unpaired two-tailed student's *t*-test, *p* = 0.1, Table 5) but a significant decreased anaerobic diversity in kwashiorkor (1.05 ± 0.98 vs. 3.1 ± 1.5, two-tailed Mann Whitney test, *p* = 0.02, Table 5). These results confirmed the specific and drastic decrease in anaerobic diversity found by culturomics.

### Proteobacteria and *Streptococcus gallolyticus* increase in kwashiorkor

**Figure 4:**
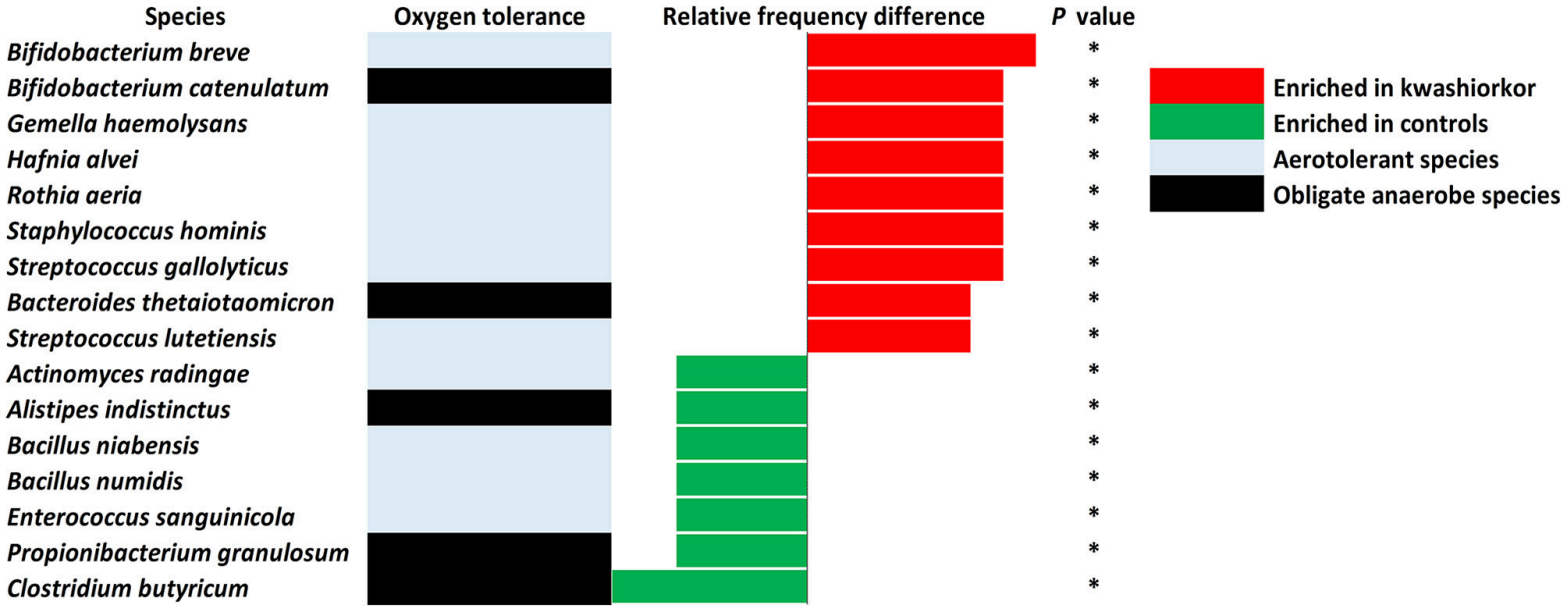
**Increased frequency of species in Kwashiorkor samples and control samples for the culturomics approach**. Each bar represents the relative frequency difference for each species with red bars representing an increased frequency in patients with kwashiorkor and green bars representing an increased frequency in controls. A majority of oxygen-tolerant species are increased in the gut of patients with kwashiorkor. *P-value ranging from 0.01 and 0.05.

With culturomics, five bacterial phyla were isolated in kwashiorkor with a majority of *Firmicutes* (208 species), followed by 56 *Proteobacteria*, 47 *Actinobacteria*, 21 *Bacteroidetes*, and 3 *Fusobacteria*. A total of 108 genera were isolated including *Clostridium* (46 species), *Bacillus* (28), *Streptococcus* (18), *Staphylococcus* (17), *Enterococcus* (13), *Paenibacillus* (11), *Lactobacillus* (13), and *Corynebacterium* (9). In control samples, only four phyla were isolated: *Firmicutes* (192 species), *Actinobacteria* (42), *Proteobacteria* (25), and *Bacteroidetes* (21). Strikingly, there was no *Fusobacteria* species. A total of 91 genera were identified among these four phyla. The most represented were *Clostridium* (45 species), *Bacillus* (30), *Paenibacillus* (18), *Streptococcus* (12), *Staphylococcus* (9), and *Lactobacillus* (8) (Table 4). A significant increase in the frequency of *Proteobacteria* was found in kwashiorkor [56/335 (17%) vs. 25/281 (9%) in controls, uncorrected two-tailed chi-square test, *p* = 0.004, Table 2]. There was no significant difference for other phyla (Table 2). At the species level, significantly enriched species in kwashiorkor (Figure 4) included *Bacteroides thetaiotaomicron, Bifidobacterium breve, Bifidobacterium catenulatum, Gemella haemolysans, Hafnia alvei, Rothia aeria, Staphylococcus hominis, Streptococcus gallolyticus*, and *Streptococcus lutetiensis*. These species are susceptible to β-lactam antibiotics (Marrie and Kwan, 1982; Moubareck et al., 2005; Stock et al., 2005; Michon et al., 2010; Carlier et al., 2015) which are used in kwashiorkor treatment (Lazzerini and Tickell, 2011; Trehan et al., 2013; Million et al., 2017a). Species such as *G. haemolysans, H. alvei, S. gallolyticus*, and *S. lutetiensis* are potentially pathogenic and have been previously associated with diarrhea, gastroenteritis, and endocarditis (Helft et al., 1993; Abbott et al., 2011; Jin et al., 2013).

The metagenomics analysis showed that in kwashiorkor samples, 2,783,881 reads assigned at a prokaryotic species level were distributed into nine phyla (*Actinobacteria, Bacteroidetes, Chloroflexi, Firmicutes, Fusobacteria, Planctomycetes, Proteobacteria, Synergistetes*, and *Verrucomicrobia*). Control samples generated 1,333,589 reads assigned at a prokaryotic species level and divided into the following eight phyla: *Actinobacteria, Bacteroidetes, Firmicutes, Fusobacteria, Lentisphaerae, Proteobacteria, Tenericutes*, and *Verrucomicrobia*. *Chloroflexi, Planctomycetes*, and *Synergistetes* were detected only in patients with kwashiorkor whereas *Lentisphaerae* and *Tenericutes* were detected only in control patients. These reads matched 589 species in the kwashiorkor group and 486 in the control group. *Proteobacteria* were also detected more frequently in kwashiorkor [131/589 (22%) vs. 75/486 (15%) in controls, uncorrected two-tailed chi square test, *p* = 0.004, Table 6] thus confirming the culturomics results. Interestingly, among *Proteobacteria*, the difference was significant only for alpha-*Proteobacteria* [22/589 (3.7%) vs. 2/486 (0.4%), *p* = 0.0002, Table 6]. *Firmicutes* were detected less frequently [319/589 (54%) vs. 296/486 (61%), *p* = 0.026, Table 6] and *Euryarchaeota* were not detected in kwashiorkor [0/589 (0%) vs. 4/486 (0.8%), *p* = 0.027, Table 6].

**Table 6:** **Comparison of the metagenomics gut bacterial diversity between children with kwashiorkor and control children**.

	**Kwashiorkor (*n* = 10)**	**Controls (*n* = 5)**	***P*-value**
**GLOBAL BACTERIAL ABUNDANCE**			
Proportion of reads assigned at the species level^a^ (mean ±*SD*)	0.95 ± 0.02	0.74 ± 0.22	0.009^b^
**PROPORTION PER PHYLUM**^c^			
*Actinobacteria*	86/589 (14%)	61/486 (12%)	0.37^d^
*Bacteroidetes*	44/589 (7%)	46/486 (9%)	0.24^e^
*Chloroflexi*	1/589 (0.2%)	0/486 (0%)	0.36^e^
*Euryarchaeota*	0/589 (0%)	4/486 (0.8%)	0.027^e^
*Firmicutes*	319/589 (54%)	296/486 (61%)	0.026^d^
*Fusobacteria*	7/589 (1.2%)	1/486 (0.2%)	0.12^e^
*Lentisphaerae*	0/589 (0%)	1/486 (0.2%)	0.90^e^
*Proteobacteria*	131/589 (22%)	75/486 (15%)	0.004^d^
*Tenericutes*	0/589 (0%)	1/486 (0.2%)	0.90^e^
*Verrucomicrobia*	1/589 (0.2%)	1/486 (0.2%)	>0.99^e^
**PROPORTION PER CLASS**			
*Actinobacteria*	69/589 (12%)	38/486 (8%)	0.033^d^
*Coriobacteriia*	16/589 (3%)	22/486 (4%)	0.109^d^
*Bacteroidia*	38/589 (6%)	46/486 (9%)	0.066^d^
*Flavobacteriia*	0/589 (0%)	4/486 (0.8%)	0.082^e^
*Sphingobacteriia*	2/589 (0.3%)	0/486 (0%)	0.59^e^
*Bacilli*	158/589 (27%)	124/486 (25%)	0.63^d^
*Clostridia*	115/589 (19%)	132/486 (27%)	0.003^d^
*Erysipelotrichia*	11/589 (2%)	14/486 (3%)	0.27^d^
*Negativicutes*	31/589 (5%)	25/486 (5%)	>0.99^d^
*Tissierellia*	4/589 (0.7%)	1/486 (0.2%)	0.50^e^
*Alphaproteobacteria*	22/589 (4%)	2/486 (0.4%)	0.0002^d^
*Betaproteobacteria*	12/589 (2%)	6/486 (1%)	0.31^d^
*Deltaproteobacteria*	5/589 (0.8%)	3/486 (0.6%)	0.94^d^
*Epsilonproteobacteria*	2/589 (0.3%)	2/486 (0.4%)	>0.99^e^
*Gammaproteobacteria*	91/589 (15%)	63/486 (13%)	0.25^d^
*Fusobacteriia*	7/589 (1%)	1/486 (0.2%)	0.12^d^
*Lentisphaeria*	0/589 (0%)	1/486 (0.2%)	0.90^e^
*Mollicutes*	0/589 (0%)	1/486 (0.2%)	0.90^e^
*Verrucomicrobiae*	1/589 (0.2%)	1/486 (0.2%)	>0.99^e^
*Chloroflexia*	1/589 (0.2%)	0/486 (0%)	>0.99^e^

*SD, Standard deviation*.

a *Result per sample*.

b *Two-tailed unpaired t-test*.

c *Number of species belonging to this taxonomical group divided by the total number of species in this group*.

d *Uncorrected two-tailed chi square test*.

e *Exact two-tailed Fisher test*.

*The proportion of reads was estimated per sample. Diversity was estimated for all samples by calculating proportions of species in each phylum or class. Firmicutes species were significantly decreased in kwashiorkor, specifically species belonging to the Clostridia class. Proteobacteria species were significantly increased in kwashiorkor, specifically species belonging to the Alphaproteobacteria class. No Euryarchaeota was found in kwashiorkor*.

**Figure 5:**
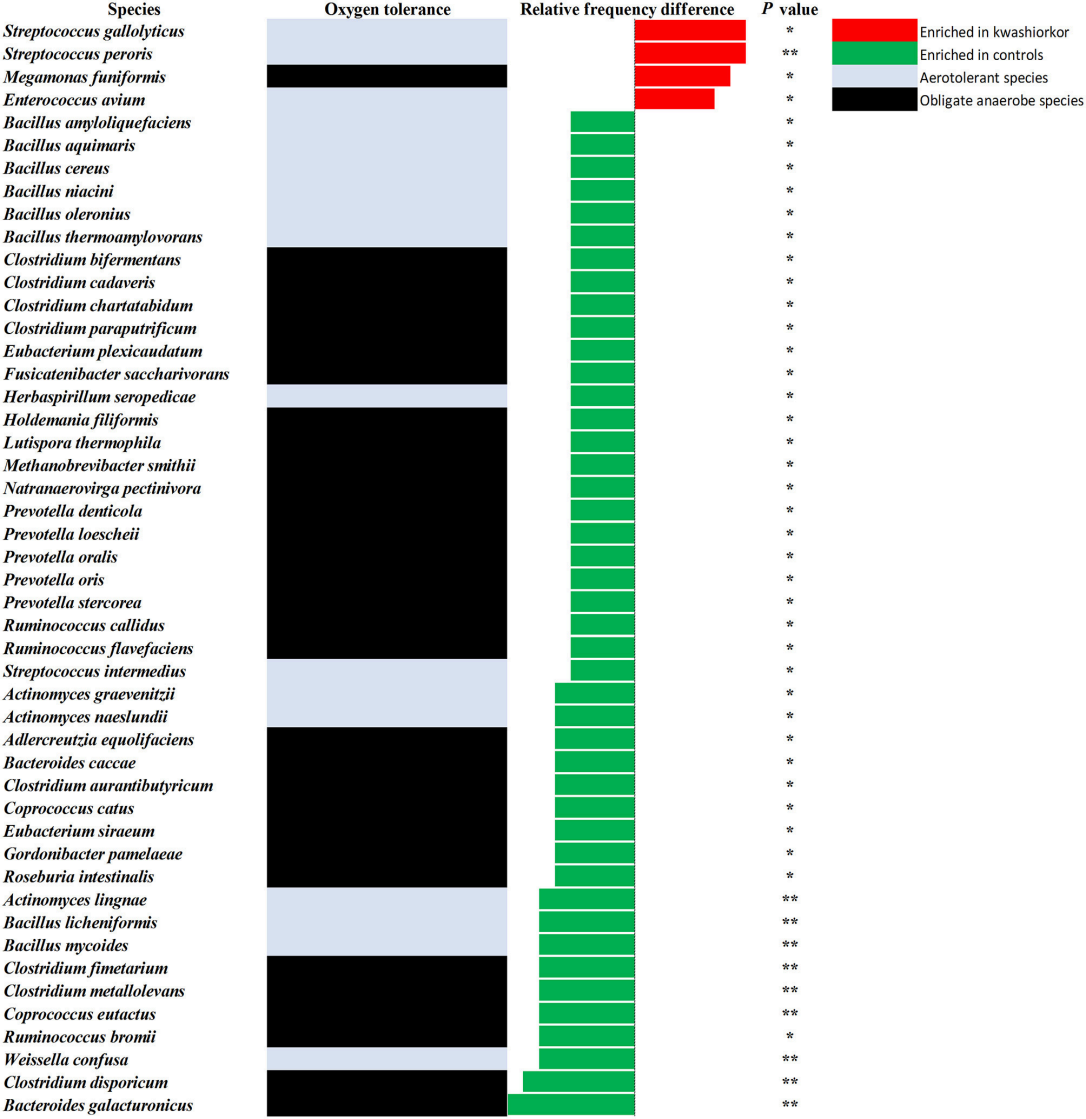
**Increased frequency of species in Kwashiorkor samples and control samples for the metagenomics approach**. Each bar represents the relative frequency difference for each species with red bars representing an increased frequency in patients with kwashiorkor and green bars representing an increased frequency in controls. Only four species were significantly increased in patients with kwashiorkor among which three were aerotolerant. *P-value ranging from 0.01 and 0.05. **P-value ranging from 0.001 and 0.01.

At the genus level, *Streptococcus* were more frequent in kwashiorkor [40/589 (6.8%) vs. 23/486 (4.7%) in controls, uncorrected two-tailed chi square, *p* = 0.029] whereas *Prevotella* and *Bacillus* were less frequent in kwashiorkor [2/589 (0.3%) vs. 14/486 (2.9%) in controls, *p* = 0.0006 and 11/589 (1.9%) vs. 28/486 (5.7%), *p* = 0.0006, respectively, Table 6]. At the species level, the two species with the largest increased frequency in kwashiorkor belong to the *Streptococcus* genus including *Streptococcus peroris* [9/10 (90%) in kwashiorkor vs. 1/5 (20%) in controls, two-tailed Barnard test *p* = 0.009] and *S. gallolyticus* [7/10 (70%) in kwashiorkor vs. 0/5 (0%) in controls, two-tailed Barnard test *p* = 0.014, Figure 5].

### Missing repertoire in kwashiorkor patients

Species identified by metagenomics and culturomics in kwashiorkor and control patients were compared and 45 species were identified only in control samples (Table 7). These species belonged overwhelmingly to the *Firmicutes* phylum (32) followed by a few species from the *Actinobacteria* (5), the *Bacteroidetes* (4), and the *Proteobacteria* (4) phyla. Among the missing repertoire, strikingly, 23 species (51%) were strictly anaerobic.

**Table 7:** **Missing microbes in Kwashiorkor identified both by culturomics and metagenomics**.

**Species**	**Obligate anaerobes**	**Phylum**	**Class**	**Order**	**Family**	**Genera**	**Possible probiotic**
*Acinetobacter lwoffii*	0	*Proteobacteria*	*Gammaproteobacteria*	*Pseudomonadales*	*Moraxellaceae*	*Acinetobacter*	No
*Alistipes indistinctus*	1	*Bacteroidetes*	*Bacteroidia*	*Bacteroidales*	*Rikenellaceae*	*Alistipes*	Yes
*Alistipes putredinis*	1	*Bacteroidetes*	*Bacteroidia*	*Bacteroidales*	*Rikenellaceae*	*Alistipes*	No
*Alistipes senegalensis*	1	*Bacteroidetes*	*Bacteroidia*	*Bacteroidales*	*Rikenellaceae*	*Alistipes*	No
*Alloscardovia omnicolens*	0	*Actinobacteria*	*Actinobacteria*	*Bifidobacteriales*	*Bifidobacteriaceae*	*Alloscardovia*	No
*Anaerostipes caccae*	1	*Firmicutes*	*Clostridia*	*Clostridiales*	*Lachnospiraceae*	*Anaerostipes*	Yes
*Arthrobacter agilis*	0	*Actinobacteria*	*Actinobacteria*	*Micrococcales*	*Micrococcaceae*	*Arthrobacter*	No
*Asaccharospora irregularis*	1	*Firmicutes*	*Clostridia*	*Clostridiales*	*Peptostreptococcaceae*	*Asaccharospora*	No
*Bacillus cereus*	0	*Firmicutes*	*Bacilli*	*Bacillales*	*Bacillaceae*	*Bacillus*	No
*Bacillus firmus*	0	*Firmicutes*	*Bacilli*	*Bacillales*	*Bacillaceae*	*Bacillus*	No
*Bacillus idriensis*	0	*Firmicutes*	*Bacilli*	*Bacillales*	*Bacillaceae*	*Bacillus*	No
*Bacillus licheniformis*	0	*Firmicutes*	*Bacilli*	*Bacillales*	*Bacillaceae*	*Bacillus*	Yes
*Bacillus niabensis*	0	*Firmicutes*	*Bacilli*	*Bacillales*	*Bacillaceae*	*Bacillus*	No
*Bacillus subtilis*	0	*Firmicutes*	*Bacilli*	*Bacillales*	*Bacillaceae*	*Bacillus*	Yes
*Bacillus thermoamylovorans*	0	*Firmicutes*	*Bacilli*	*Bacillales*	*Bacillaceae*	*Bacillus*	No
*Bacteroides salyersiae*	1	*Bacteroidetes*	*Bacteroidia*	*Bacteroidales*	*Bacteroidaceae*	*Bacteroides*	Yes
*Bifidobacterium adolescentis*	1	*Actinobacteria*	*Actinobacteria*	*Bifidobacteriales*	*Bifidobacteriaceae*	*Bifidobacterium*	Yes
*Clostridium amygdalinum*	1	*Firmicutes*	*Clostridia*	*Clostridiales*	*Lachnospiraceae*	*Lachnoclostridium*	No
*Clostridium cadaveris*	1	*Firmicutes*	*Clostridia*	*Clostridiales*	*Clostridiaceae*	*Clostridium*	No
*Clostridium glycolicum*	1	*Firmicutes*	*Clostridia*	*Clostridiales*	*Peptostreptococcaceae*	*Terrisporobacter*	Yes
*Clostridium hylemonae*	1	*Firmicutes*	*Clostridia*	*Clostridiales*	*Clostridiaceae*	*Clostridium*	Yes
*Clostridium neonatale*	1	*Firmicutes*	*Clostridia*	*Clostridiales*	*Clostridiaceae*	*Clostridium*	No
*Clostridium oroticum*	1	*Firmicutes*	*Clostridia*	*Clostridiales*	*Lachnospiraceae*	*Lachnoclostridium*	No
*Clostridium paraputrificum*	1	*Firmicutes*	*Clostridia*	*Clostridiales*	*Clostridiaceae*	*Clostridium*	No
*Clostridium saccharolyticum*	1	*Firmicutes*	*Clostridia*	*Clostridiales*	*Clostridiaceae*	*Clostridium*	No
*Clostridium sordellii*	1	*Firmicutes*	*Clostridia*	*Clostridiales*	*Peptostreptococcaceae*	*Peptoclostridium*	No
*Dialister pneumosintes*	1	*Firmicutes*	*Negativicutes*	*Selenomonadales*	*Veillonellaceae*	*Dialister*	No
*Enterococcus dispar*	0	*Firmicutes*	*Bacilli*	*Lactobacillales*	*Enterococcaceae*	*Enterococcus*	No
*Faecalitalea cylindroides*	1	*Firmicutes*	*Erysipelotrichia*	*Erysipelotrichales*	*Erysipelotrichaceae*	*Faecalitalea*	No
*Gemella sanguinis*	0	*Firmicutes*	*Bacilli*	*Bacillales*	*Bacillales Family XI Incertae Sedis*	*Gemella*	No
*Intestinimonas butyriciproducens*	1	*Firmicutes*	*Clostridia*	*Clostridiales*	*Unclassified clostridiales*	*Intestinimonas*	Yes
*Lactobacillus parabuchneri*	0	*Firmicutes*	*Bacilli*	*Lactobacillales*	*Lactobacillaceae*	*Lactobacillus*	Yes
*Lactobacillus perolens*	0	*Firmicutes*	*Bacilli*	*Lactobacillales*	*Lactobacillaceae*	*Lactobacillus*	Yes
*Lactobacillus vaccinostercus*	0	*Firmicutes*	*Bacilli*	*Lactobacillales*	*Lactobacillaceae*	*Lactobacillus*	Yes
*Micrococcus lylae*	0	*Actinobacteria*	*Actinobacteria*	*Micrococcales*	*Micrococcaceae*	*Micrococcus*	No
*Neisseria flavescens*	0	*Proteobacteria*	*Betaproteobacteria*	*Neisseriales*	*Neisseriaceae*	*Neisseria*	No
*Pantoea septica*	0	*Proteobacteria*	*Gammaproteobacteria*	*Enterobacteriales*	*Enterobacteriaceae*	*Pantoea*	No
*Paraclostridium bifermentans*	1	*Firmicutes*	*Clostridia*	*Clostridiales*	*Peptostreptococcaceae*	*Paraclostridium*	No
*Slackia exigua*	1	*Actinobacteria*	*Coriobacteriia*	*Eggerthellales*	*Eggerthellaceae*	*Slackia*	No
*Staphylococcus haemolyticus*	0	*Firmicutes*	*Bacilli*	*Bacillales*	*Staphylococcaceae*	*Staphylococcus*	No
*Staphylococcus hominis*	0	*Firmicutes*	*Bacilli*	*Bacillales*	*Staphylococcaceae*	*Staphylococcus*	No
*Streptococcus vestibularis*	0	*Firmicutes*	*Bacilli*	*Lactobacillales*	*Streptococcaceae*	*Streptococcus*	No
*Sutterella wadsworthensis*	1	*Proteobacteria*	*Betaproteobacteria*	*Burkholderiales*	*Sutterellaceae*	*Sutterella*	No
*Veillonella dispar*	1	*Firmicutes*	*Negativicutes*	*Selenomonadales*	*Veillonellaceae*	*Veillonella*	No
*Weissella confusa*	0	*Firmicutes*	*Bacilli*	*Lactobacillales*	*Leuconostocaceae*	*Wesseila*	Yes

For each of these species, we searched through the literature to find a possible probiotic use. Twelve species, nine *Firmicutes*, two *Bacteroidetes*, and one *Actinobacteria* were found to have possible probiotic features or were representative of a healthy flora: *Alistipes indistinctus, Anaerostipes caccae, Bacillus licheniformis, Bacillus subtilis, Bacteroides salyersiae, Bifidobacterium adolescentis, Intestinimonas butyriciproducens, Lactobacillus perolens, Lactobacillus parabuchneri, Lactobacillus vaccinostercus, Terrisporobacter glycolicus*, and *Weissella confusa* (Table 8). Probiotic features included short chain fatty acid production, antioxidant metabolism, and antibacterial potential. Each of these species was isolated in our control group of samples and is readily available in the CSUR collection of our laboratory.

**Table 8:** **Potential probiotics identified by culturomics and metagenomics and their possible function**.

**Species**	**Obligate anaerobe^a^**	**Phylum**	**Function**
*Alistipes indistinctus*	1	*Bacteroidetes*	Common member of the gut microbiota of healthy humans (Nagai et al., 2010)
*Anaerostipes caccae*	1	*Firmicutes*	Common member of the gut microbiota of healthy humans (Maukonen and Saarela, 2015)
*Bacillus licheniformis*	0	*Firmicutes*	Antibacterial potential (Shobharani et al., 2015)
*Bacillus subtilis*	0	*Firmicutes*	Antibacterial potential (Hong et al., 2009)
*Bacteroides salyersiae*	1	*Bacteroidetes*	Mutualistic association with *T. glycolicus* for polysaccharides fermentation (Hunger et al., 2011)
*Bifidobacterium adolescentis*	1	*Actinobacteria*	Common member of the gut microbiota in healthy breastfed infants (Jost et al., 2015)
*Intestinimonas butyriciproducens*	1	*Firmicutes*	Butyrate production (Engels et al., 2016)
*Lactobacillus parabuchneri*	0	*Firmicutes*	Common member of the gut microbiota of healthy breastfed infants (Jost et al., 2015)
*Lactobacillus perolens*	0	*Firmicutes*	Common member of the gut microbiota of healthy breastfed infants (Jost et al., 2015)
*Lactobacillus vaccinostercus*	0	*Firmicutes*	Common member of the gut microbiota of healthy breastfed infants (Jost et al., 2015)
*Terrisporobacter glycolicus*	1	*Firmicutes*	Mutualistic association with acetogenic *Bacteroides* for polysaccharides fermentation (Jost et al., 2015)
*Weissella confusa*	0	*Firmicutes*	Antioxidant metabolism (Zhang et al., 2014)

a *1, obligate anaerobic species. 0, facultative anaerobe or aerobic species*.

## Discussion

In this study, we identified 45 living, viable and cultivable bacterial species and confirmed by metagenomics to be present in controls but missing in the feces of children with kwashiorkor. Among these, an analysis of the literature identified 12 species that met the criteria desirable for a probiotic mixture to be used as defined in our introduction; viable in healthy gut environment, do not produce any toxic product, symbiotic with healthy resident microbiota, producing butyrate and/or propionate and anti-pathogenic bacteriocins, promotes gut environment characteristic of good health. Conversely, characteristics of the kwashiorkor-associated gut microbiota included a depletion of the hitherto unknown, global and anaerobic diversity, and enrichment in potentially pathogenic and oxidative stress-resistant *Fusobacteria* and *Proteobacteria*.

*Streptococcus gallolyticus* was the only bacterial species to be associated with kwashiorkor in the present African study both by culturomics and metagenomics. This finding is not random as *S. gallolyticus* was one of the three species enriched in SAM while 31 other species were enriched in controls in the largest metagenomics study to date performed in Asia (Subramanian et al., 2014). This is clinically relevant as *S. gallolyticus* is one of the human pathogenic bacteria with the strongest association with colon cancer and endocarditis (Rusniok et al., 2010; Amado et al., 2015; Butt et al., 2016). Among the 117 Streptococcus validated species (http://www.bacterio.net/streptococcus.html), only *S. gallolyticus* has such a pathogenic potential. The species-level resolution of the characterization of the gut microbiota alteration associated with SAM is also critical as a species and strain specificity of probiotics effect on weight regulation has previously been demonstrated (Million et al., 2012; Million and Raoult, 2013). As *S. gallolyticus* strains are always susceptible to amoxicillin, this is a new argument to confirm the inclusion of amoxicillin in the standard protocols for severe acute malnutrition as recently demonstrated by a meta-analysis (Million et al., 2017a). This also suggests that future probiotic mixtures to treat SAM should inhibit *S. gallolyticus*.

The overabundance of *Proteobacteria* was observed in other studies using both cultivation and metagenomics approaches with an increase of the pathogenic *Shigella, Edwardsiella*, and *Salmonella* by culture in the gut of malnourished children (Million et al., 2017b). Many pathogenic species belonging to the *Proteobacteria* and *Fusobacteria* phyla are sensitive to large spectrum β-lactam antibiotics, such as ampicillin and cephalosporins (Stock et al., 2005; Roberts et al., 2006; Poulsen et al., 2012; Nomoto et al., 2013) showing how these antibiotics can drastically improve recovery and mortality rates in malnourished patients (Million et al., 2016). Further, well-designed studies are needed to confirm preliminary results suggesting that cephalosporins, with a broader spectrum on *Proteobacteria* species, are better than amoxicillin in the routine management of severe acute malnutrition (Trehan et al., 2013).

As determined by the strict application of the WHO criteria to define child growth standards (WHO and UNICEF, 2009), each of our patients with kwashiorkor was a textbook case and each of our controls was a healthy child under 5 years of age. Cases and controls included in this study originated from two different geographic locations thus allowing a generalization of the results. Moreover, every sample was analyzed using the same protocol for metagenomics with a specific DNA extraction protocol (Dridi et al., 2009) and used with or without prior deglycosylation of the sample (Angelakis et al., 2016); the same 18 culture conditions were also used for culturomics.

The “microbial culturomics” method, whose efficacy in exploring the gut microbiota is no longer to be proven (Lagier et al., 2016), presents like other culture approaches (Lagkouvardos et al., 2016) the tremendous advantage over metagenomics to exclude the huge number of ingested bacteria living in the diet (Lang et al., 2014) but killed in the upper intestine by nitric oxide, acidic environment, and bile salts and to provide a physical collection of strains on which further analysis can be carried out. Metagenomics studies are the preferential technique for the exploration of the gut microbiota diversity but have a very low reproducibility among studies probably due to the differences in sampling, sample conservation, DNA extraction protocol, sequencing method, and data analysis strategy (Maukonen and Saarela, 2015). Moreover, culture approaches allow the extension of the gut microbiota known diversity and functions (Lagkouvardos et al., 2016). A discordance between microbial culturomics and metagenomics has also been highlighted in several studies (Lagier et al., 2012, 2015b; Dubourg et al., 2014). Conversely, congruence between the two techniques is observed at the global level with similar loss of overall, unknown, and anaerobic diversity alongside an enrichment of *Proteobacteria*.

The infant gut microbiota has been shown to drive the weight gain and skeletal growth with a species- and even strain-specific effect (Blanton et al., 2016; Schwarzer et al., 2016). Culturomics associated with mass spectrometry is currently the most accurate technique to identify species and strains that could serve as probiotics (Hill et al., 2014). The 12 bacterial species that met our pre-defined criteria desirable for a probiotic mixture to be used in SAM (Table 8) are commensal of the gut microbiota of healthy infants (Bäckhed et al., 2015; Jost et al., 2015) and adults (Maukonen and Saarela, 2015; Engels et al., 2016), produce antimicrobial compounds (Hong et al., 2009; Shobharani et al., 2015), contribute to the antioxidant metabolism (Zhang et al., 2014), and contribute to a mutualistic association with commensals of the gut microbiota (Hunger et al., 2011). *W. confusa* (Table 8) was described as a probiotic fulfilling all the aforementioned conditions (Nam et al., 2002; Lee et al., 2012; Goh and Philip, 2015; Malik et al., 2016); so were *B. licheniformis* and *B. subtilis* (Table 8) in animals (Gaggìa et al., 2010). Administration of these potential probiotics associated with *M. smithii* can replace fecal transplant as a way to restore a healthy gut microbiota in malnourished children and provide an easy addition to current SAM treatment as opposed to fecal transplantation (Khoruts et al., 2015).

The depletion of the hitherto unknown and anaerobic diversity most likely represents a depletion of micronutrients (minerals, prebiotics, anti-oxidants) and a loss of the reduced environment which both are culture requirements for most strict anaerobes. The depletion of anaerobic species in the gut microbiota of patients with kwashiorkor is here observed for the first time using a culture-based approach, except for a study by Mata et al. in 1972, realized at a low scale, which reported a decrease in anaerobic species in the proximal gut and the feces of malnourished children (Mata et al., 1972). This depletion in anaerobic species has previously been linked to an oxidized environment, an absence of *M. smithii*, the growth of which depends heavily on hydrogen-producing species and a minimal to no oxygen tension in the environment (Samuel and Gordon, 2006; Dione et al., 2016; Million et al., 2016). Consistently, a shift in pH-values has previously been associated with an abnormal flora (Donders et al., 2016) as observed in patients with kwashiorkor. The low inter-individual variability observed in patients with kwashiorkor is probably due to a similarly low variability in the diet of patients with kwashiorkor. In fact, the diet of children in regions with a high SAM prevalence is often made of starchy food like cassava, yam, maize, and banana among others and characterized by a low intake in animal products (Kismul et al., 2014).

Of the 30 species enriched in controls (Figure 5) and identified only by metagenomics, three were particularly associated with a beneficial effect for the host in the literature (Takagaki and Nanjo, 2015; Ze et al., 2015; Tamanai-Shacoori et al., 2017). These three species namely *Roseburia intestinalis, Ruminococcus broomii*, and *Adlercreutzia equolifaciens* can also be beneficial in the restoration of a healthy flora, short chain fatty acid production, and antioxidants metabolism. These species are extremely oxygen-sensitive bacteria requiring specific culturomics strategies using fresh samples and antioxidants in the transport medium to enhance growth and protect them from oxygen (Dione et al., 2016). Such species could have been destroyed during the transport to our laboratory and should be also considered as potential probiotics to treat SAM. However, as a probiotic mixture to treat SAM should be relatively easy to produce and transport, such fastidious bacteria should be considered only if a simpler probiotic mixture is not efficient. Even if the 18 culture conditions of the culturomics approach have been selected to standardize the process (Lagier et al., 2016), addition of a culture medium as YCFAG could usefully be added to our repertoire of culture conditions to obtain extremely oxygen-sensitive bacteria such as *Faecalibacterium prausnitzii*.

The number of patients who gave samples is small, however similar results have been found from larger studies and from elsewhere (Subramanian et al., 2014). Nevertheless, the two countries studied are both situated in the Sahel (desert territories) where kwashiorkor is uncommon. Sampling needs to be extended to countries where kwashiorkor is the dominant form of severe acute malnutrition and also to children who have both marasmus and kwashiorkor. The patients have not been well-characterized clinically or biochemically although they satisfied the WHO criteria for kwashiorkor. Such children have a wide spectrum of severity and different degrees of immunological and physiological defects which need to be related to changes in their gut microbiota. In Niger, age and sex of the patients were not recorded and their controls samples were not taken contemporaneously. In Senegal, full anthropometric and clinical data were recorded and the samples from the cases and controls were taken at the same time.

We are not able to ascertain that the mothers have not given antibiotics to the children prior to attending the hospital as antibiotics are freely available for purchase in the market of such countries. Similarly, patients frequently consult a traditional practitioner prior to attending Western medical facilities. It is unknown what the effect of the traditional potions may have on the gut microbiota.

The organisms which we focused on were those which we grew by culturomics and identified by metagenomics. Other organisms which we grew may also be useful probiotics species. The culture conditions that we used were not exhaustive but did include the conditions which grew most species and were practicable. Extending the culturomics to additional culture conditions may improve the overlap in the two conditions and allow us in the future to identify more useful species. The characteristics we use to judge the potential usefulness of a bacterium as a probiotic are not established. For example, the question of the sensitivity to bile acids is not clear. A bacterium that metabolizes cholic acid to lithocholic acid would be detrimental as lithocholate is hepatotoxic and bile acids are required for absorption of fat soluble vitamins and lipids. Alternatively, sensitivity to bile salts may prevent useful bacteria from establishing themselves in the lower intestine.

In this study, we identified bacteria that met the characteristics of potential probiotics. In order to test the efficacy of these possible probiotics, we aimed at developing an experimental model with axenic mice on which this cocktail of probiotics would be tested. All studies concerning the gut microbiota of SAM patients are passive, exploratory studies. The value of culturomics is that we are able to ensure that identified bacteria are viable and can be grown in culture so that a clearly defined bacterial mixture can be administered to the patients whereas fecal transplant is likely to vary depending on the particular bacteria mixture of the donor. Each donor will be different. Efficacy of fecal microbiota transplantation to prevent death was recently demonstrated in the elderly in the context of the ongoing *Clostridium difficile* outbreak (Lagier et al., 2015a), and multispecies probiotics derived from the feces of healthy humans was shown to be an equivalent to fecal microbiota transplantation in the same context (Petrof et al., 2013). It is time to apply the experience acquired thanks to the elderly to take care of humanity's most vulnerable children.

## Author contributions

Conceptualization: JCL and DR. Methodology: SK and DR. Validation: MM and DR. Formal analysis: MTA, MM, and DR. Investigation: MTA, SIT, DM, and CR. Resources: SB, DA, CS, and AD. Data curation: DB, AC, and JD. Writing-Original draft: MTA, MM, and DR. Writing-Review and Editing: MTA, MM, JCL, MG, and DR. Visualization: MTA and MM. Supervision: MM, SK, JCL, BAD, and DR. Project Administration: PP and DR. Funding Acquisition: DR.

### Conflict of interest statement

The authors declare that the research was conducted in the absence of any commercial or financial relationships that could be construed as a potential conflict of interest. The reviewer ES-T and handling Editor declared their shared affiliation, and the handling Editor states that the process nevertheless met the standards of a fair and objective review.

## Abbreviations

CSUR: Collection de Souches de l'Unité des Rickettsies
HAZ: Height-for-age z-score
HMO: Human Milk Oligosaccharides
MALDI TOF MS: Matrix Assisted Laser Desorption/Ionization Mass Spectrometry
MUAC: Mid-Upper Arm Circumference
OTU: Operational Taxonomic Unit
RUTF: Ready-To-Use Therapeutic Food
SAM: Severe Acute Malnutrition
SD: Standard Deviation
WAZ: Weigth-for-Age z-score
WHZ: Weigth-for-Height z-score.
