# Supplementary material for: Gut Bacteria Missing in Severe Acute Malnutrition, Can We Identify Potential Probiotics by Culturomics?
Source: Front Microbiol. 2017 May 23;8:899. doi: 10.3389/fmicb.2017.00899 (PMC5440526; doi:10.3389/fmicb.2017.00899)
Supplement: Supplementary file 1 [file Table1.DOCX]

**Supplementary Table 1. List of samples according to geographic origin, sex and age.**

| **Sample name** | **Origin** | **Sex** | **Age** |
| --- | --- | --- | --- |
| Kwashiorkor 1 | Niger | NA | NA |
| Kwashiorkor 8 | Niger | NA | NA |
| Kwashiorkor 12 | Niger | NA | NA |
| Kwashiorkor 14 | Niger | NA | NA |
| Kwashiorkor 01 | Senegal | Female | 7 months old |
| Kwashiorkor 02 | Senegal | Female | 49 months old |
| Kwashiorkor 04 | Senegal | Female | 4 months old |
| Kwashiorkor 05 | Senegal | Male | 12 months old |
| Kwashiorkor 06 | Senegal | Male | 6 months old |
| Kwashiorkor 010 | Senegal | Male | 2.2 months old |
| Control N6 | Niger | Female | 7 months old |
| Control N12 | Niger | Female | 44 months old |
| Control S05 | Senegal | Male | 28.6 months old |
| Control S07 | Senegal | Female | 38 months old |
| Control S50/04 | Senegal | Female | 8 months old |

NA: non-available data
