# Supplementary material for: Gut Bacteria Missing in Severe Acute Malnutrition, Can We Identify Potential Probiotics by Culturomics?
Source: Front Microbiol. 2017 May 23;8:899. doi: 10.3389/fmicb.2017.00899 (PMC5440526; doi:10.3389/fmicb.2017.00899)
Supplement: Supplementary file 2 [file Table2.docx]

**Supplementary Table 2. List of primers and probes used in our study.**

| Organism | Assay | Primer/probe name  and sequence (5’-3’) | Dye |
| --- | --- | --- | --- |
| *Methanobrevibacter*  *smithii*^1^ | 16S rDNA | Smit.16S-740F, CCGGGTATCTAATCCGGTTC  Smit.16S-862R, CTCCCAGGGTAGAGGTGAAA  Smit.16SFAM,CCGTCAGAATCGTTCCAGTCAG | FAM (MGB) |
| 16S rRNA amplification^2^ | 16S rRNA | fD1, AGAGTTTGATCATGGCTCAG  rP2, ACGGCTACCTTGTTACGACTT |  |
| 16S rRNA sequencing^2^ | 16S rRNA | 357F, TACGGGAGGCAGCAG  357R, CTGCTGCCTCCCGTA  536F, CAGCAGCCGCGGTAATAC  536R, GTATTACCGCGGCTGCTG  800F, ATTAGATACCCTGGTAG  800R, CTACCAGGGTATCTAAT  1050F, TGTCGTCAGCTCGTG  1050R,CACGAGCTGACGACA |  |

^1^Dridi, B., Henry, M., El Khéchine, A., Raoult, D., and Drancourt, M. (2009). High prevalence of *Methanobrevibacter smithii* and *Methanosphaera stadtmanae* detected in the human gut using an improved DNA detection protocol. PloS One *4*, e7063. doi: 10.1371/journal.pone.0007063. ^2^Adékambi, T. & Drancourt, M. (2004). Dissection of phylogenetic relationships among 19 rapidly growing *Mycobacterium* species by 16S rRNA, hsp65, sodA, recA and rpoB gene sequencing. Int J Syst Evol Microbiol 54, 2095–2105. doi: 10.1099/ijs.0.63094-0
